# Comparison of Humphrey MATRIX and Swedish interactive threshold algorithm standard strategy in detecting early glaucomatous visual field loss

**DOI:** 10.4103/0301-4738.49395

**Published:** 2009

**Authors:** Raju Prema, George Ronnie, Arvind Hemamalini, Ramesh Sathyamangalam Ve, Mani Baskaran, Lingam Vijaya

**Affiliations:** Glaucoma Project, Vision Research Foundation, Sankara Nethralaya, Chennai, India

**Keywords:** Frequency doubling perimetry, glaucoma, humphrey MATRIX, Swedish interactive threshold algorithm standard, visual field

## Abstract

**Purpose:**

The aim of this study was to compare the Humphrey MATRIX visual field (frequency doubling technology threshold) and Swedish interactive threshold algorithm (SITA) standard strategy white on white perimetry in detecting glaucomatous visual field loss.

**Material and Methods:**

Twenty-eight adult subjects, diagnosed to have glaucoma at a tertiary eye care hospital, who fulfilled the inclusion criteria, were included in this prospective study. All subjects underwent a complete ophthalmic examination. Subjects with glaucomatous optic disc changes underwent repeat perimetric examination on the same day with the Humphrey visual field analyzer (HFA II) and Humphrey MATRIX, the order of testing being random. Only reliable fields, where the HFA results corresponded to the disc changes were considered for analysis. A cumulative defect depth in each hemifield in both HFA and MATRIX reports was calculated.

**Results:**

Thirty-seven eyes of 24 subjects had reliable fields corresponding to optic disc changes. The mean age of the subjects was 56 ± 12 years. There were 12 males and 12 females. The test duration was significantly less on the MATRIX, mean difference in test duration was −81 ± 81.3 sec (p < 0.001). The mean deviation and the pattern standard deviation between the two instruments showed no significant difference (p = 0.55, p = 0.64 respectively) and a positive correlation coefficient of 0.63 and 0.72 respectively. Poor agreement was found with the glaucoma hemifield test.

**Conclusion:**

The Humphrey MATRIX takes less time in performing the test than SITA Standard and shows good correlation for mean deviation and pattern standard deviation. However, the glaucoma hemifield test showed poor agreement. The Humphrey MATRIX diagnoses were similar to established perimetric standards.

Glaucoma is a chronic disease that causes optic nerve damage and visual field loss. The goal of perimetry in glaucoma is to establish accurate estimates of visual field sensitivity, ideally keeping the test time to a minimum and having good reproducibility for diagnostic evaluation.

The frequency doubling technique (FDT) perimetry has been shown to perform well in detecting glaucomatous visual field loss.[1–3] The FDT perimeter tests fewer locations and therefore has a shorter duration than conventional white on white perimetry.[4] The Humphrey MATRIX (Welch Allyn, Skaneateles Falls, NY, USA and Carl Zeiss Meditec, Dublin, CA, USA) is a second-generation FDT perimeter.[5–7] MATRIX has a smaller stimulus size (5^°^),the test permits 69 visual field locations similar to 76 points on Humphrey field analyzer (HFA 30–2 program), thus providing greater detail of spatial distribution of visual field loss.[5–7]

Swedish Interactive threshold algorithm (SITA) Standard is increasingly being used as a conventional strategy for visual field testing. The Humphrey MATRIX and SITA Standard are two perimetic techniques with different testing strategies. The Matrix uses Zippy estimation of sequential testing (ZEST).[5,8–10] Studies on MATRIX have shown it to be faster than conventional HFA tests and it has been reported to detect earlier defects than white on white perimetry.[10–12] These studies have mostly used the global indices (Mean deviation-MD and Pattern standard deviation-PSD) to grade the severity of disease and to assess the diagnostic accuracy of MATRIX.[10–12] The MD and PSD calculated on two different visual field platforms are likely to be different because the techniques assess potentially different psychophysical parameters and use different testing protocols.

We aimed to compare the test indices and the cumulative defect depth obtained with the MATRIX program and the SITA Standard program in subjects with early and moderate glaucoma. The cumulative defect depth would provide information about the regional correspondence of visual field defects detected by the two techniques.

## Materials and Methods

Persons diagnosed to have glaucoma at our glaucoma clinic were considered for inclusion. Subjects who were above the age of 35 years, with a best corrected visual acuity of 20/60 or better, having no significant cataract and any other retinal and/or corneal pathology, who were willing to participate in this study were recruited. Written informed consent was obtained from all subjects and the study was performed in accordance with the tenets of the Declaration of Helsinki. The institutional review board, Vision Research Foundation, Chennai approved the study. All subjects underwent a complete ophthalmic examination including measurement of best corrected visual acuity using the modified ETDRS chart (Light House Low Vision Products, New York, NY, USA), slit-lamp examination, applanation tonometry, fundus examination using an indirect ophthalmoscope and disc evaluation with 78 diopter (D) at the slit-lamp.

Subjects were diagnosed to have glaucoma based on their clinical history, intraocular pressure (IOP) greater than 21mmHg and glaucomatous disc damage with previous corresponding visual field defects on HFA 30–2 (SITA Standard). Correspondence between optic disc and visual field defects was assessed based on the presence of field defects in the hemifield corresponding to the expected area of damage on the clinical assessment of the optic disc.

**Figure 1:**
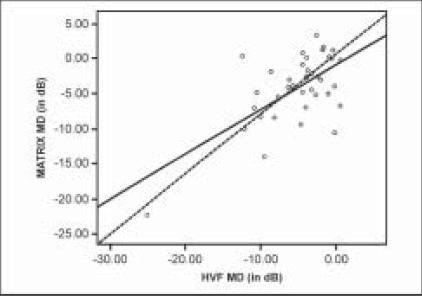
Reports of reliable SITA std and humphrey MATRIX tests showing similar visual field defects

Recruited subjects underwent a repeat perimetric examination with both the HFA and Humphrey MATRIX performed on the same day. Only these results were considered for analysis. The testing strategy used on the HFA was SITA Standard 30–2 (HFA II) (750 I series, Carl Zeiss Meditec, Dublin, CA, USA) and 30–2 FDT threshold on the MATRIX, which uses a ZEST algorithm.[9] Both tests were conducted on the same day, and the order of testing by the two perimetric methods was randomized based on computer-generated random numbers. All subjects have had previous experience with the SITA Standard and the older version of FDT-N30 full threshold strategy. Subjects performed the FDT MATRIX 30–2 program for the first time, thus a demonstration of the test was given prior to FDT MATRIX test. Only eyes with reliable fields on both the HFA and the Humphrey MATRIX were considered for analysis [Fig. 1]. The reliability criteria for both HFA and MATRIX were less than 20% fixation errors and less than 33% false positive and false negative errors. Unreliable fields, rim artifacts, classic cloverleaf patterns and visual field with advanced damage approaching fixation or showing split fixation were excluded from analysis.

Subjects with glaucomatous disc changes and reliable visual fields corresponding to the optic nerve head (ONH) findings (hemifield defects that correspond to the neural retinal rim defect) were included. Visual field defects (on SITA Standard) that satisfied at least two of three Anderson's criteria[13] were included for analysis. Glaucoma was classified as early, moderate or severe based on the Hodapp Anderson Parish (HAP)[14] classification system. If there was generalized loss of sensitivity on the glaucoma hemifield test (GHT), the other two Anderson's criteria had to be positive for inclusion.

A cumulative defect depth (a total score of the actual numerical deviations, taking into account the negative and positive deviation, seen on the pattern deviation plot) was calculated for the superior and inferior hemifield from the pattern deviation plot in both HFA and MATRIX reports. The four additional points tested on the HFA (9 degrees on the extremities of both the horizontal and vertical midlines) were excluded from this calculation. Statistical analysis was performed using SPSS Version 13, all parametric values were compared using paired sample t test. The statistical significance was considered at *P* < 0.05. The global indices and proportion defect in hemifields were compared using Pearson's correlation coefficient statistic. The agreement for GHT classifications between the perimetry techniques was assessed using the weighted kappa statistic.

## Results

**Figure 2:**
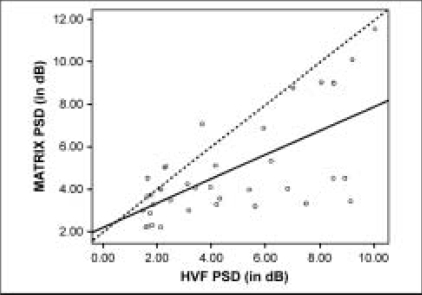
The Mean Deviation (MD) showed a positive correlation (r = 0.6) between SITA standard and MATRIX. The regression equation for linear trend was: MATRIX MD = −0.85 + 0.64 × HVF MD, R2: 0.41

**Figure 3:**
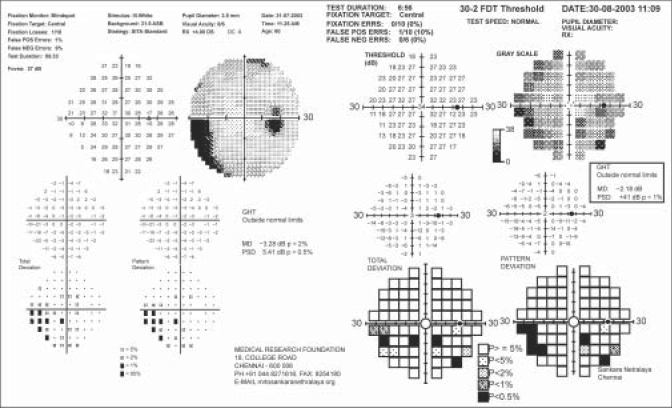
The pattern standard deviation (PSD) showed a positive correlation (r = 0.7) between SITA standard and MATRIX. The regression equation for linear trend was: MATRIX PSD = 2.22 + 0.57 × HVF PSD, R2: 0.45

Thirty-seven (24 subjects) of 47 eyes (28 subjects) had reliable fields that corresponded to the optic disc changes. Data from 10 eyes were excluded from analysis, due to unreliable perimetric tests. Twenty-nine of the 37 eyes had early glaucoma and eight eyes had moderate glaucoma as classified with the HAP. There were 12 males and 12 females. The mean age of the subjects was 56 ± 12 years. Performance was reliable in the right eye for four subjects, in the left eye for seven subjects and in both the eyes in 13 subjects. Test duration was significantly longer with HFA (T(37) = 6.34, p<0.001) Table 1. The mean difference in test duration was −81 ± 81.3 sec (range, −37 to 317 sec). There was no significant difference noted in either the MD or PSD between the two instruments (Paired sample t test: T(37) = −1.48, p = 0.55, T(37) = 0.22, p = −0.71 respectively) Table 1. The MD [Fig. 2] and the PSD [Fig. 3] between the two instruments showed a significant positive correlation coefficient of 0.6 (95% CI: 0.40 to 0.79) and 0.7 (95% CI: 0.44 to 0.81) respectively. The cumulative defect depth showed a significant positive correlation of 0.7 (95% CI: 0.45 to 0.83) in the superior quadrant and 0.4 (95% CI: 0.17 to 0.61) in the inferior quadrant (Pearson's correlation coefficient: p = 0.045 and p = 0.334 respectively). The MATRIX showed denser defect depths in both hemifields. The cumulative threshold values on the MATRIX were lower by a mean value of 57.5 dB in the superior and 26.1 dB in the inferior hemifield.

**Table 1:** Comparison of global indices and time duration between humphrey MATRIX and SITA standard strategy

	SITA std mean ± sd	Matrix mean ± sd	*P* value
Time duration (sec)	478 ± 82.72	403 ± 20.40	<0.0001
Mean Deviation	−3.82 ± 4.85	−4.38 ± 6.12	0.45
Pattern Standard Deviation	4.28 ± 3.51	4.42 ± 1.80	0.78

SITA Std: Swedish interactive threshold algorithm Standard, HVF: Humphrey visual field, SD: standard deviation, statistical significance considered at p < 0.05

Poor agreement was seen with the GHT (weighted kappa = 0.180)) between the two instruments Table 2. The GHT was further grouped as normal (within normal limit), and abnormal GHT (borderline, generalized reduced sensitivity (GRS) and outside normal limits). The visual field reports with GRS were not included to assess agreement. Fifteen reports had normal GHT on HFA, of which 13 (87%) had abnormal GHT on MATRIX and of the 22 abnormal GHT on HFA, 17 (77%) had abnormal GHT on MATRIX. There was no statistically significant difference in the proportion of abnormal GHTs between both groups (Chi square test: p = 0.07).

**Table 2:** Comparison of GHT between humphrey MATRIX and SITA standard strategy

GHT	SITA std	Matrix
Within Normal Limits	15	7
General Reduction in Sensitivity	4	2
Borderline	8	8
Outside normal limits	10	20

GHT: Glaucoma hemifield test, SITA Std: Swedish interactive threshold algorithm Standard, HVF: Humphrey visual field

## Discussion

Standard automated perimetry (SAP), which measures differential light sensitivity, is considered the gold standard of perimetry in diagnosing glaucoma. However, SAP is known to pick up glaucomatous defects only after 20–40% of the ganglion cells have been lost.[15] There is a need for techniques that could pick up damage earlier. Frequency doubling perimetry (FDP) is a newer technique that measures low spatial contrast sensitivity. The target used in the MATRIX is similar to the FDT and hence is to pick up earlier visual field defects than the SAP. Any new perimetric technique, however, would have to be validated against the gold standard before being considered for use in the diagnosis of glaucoma. Direct comparison of target thresholds between the two techniques is not possible due to variability in the dB units. The FDP uses a proprietary method to compute sensitivity; a 1dB change in FDP is approximately equal to 0.05 log unit change in Michelson contrast. In HFA the threshold sensitivity is reported in terms of log ratio between the maximum stimulus and the threshold stimulus, with 1 dB equal to 0.10 log unit change in Weber contrast.[16] The Humphrey SITA standard and FDP both use different testing strategies to detect threshold, they also measure different psychophysical attributes. We used cumulative defect depth, as a relative difference to a normative database could be expected to be similar in both cases, especially in those with significant pathology. Using single-point correspondence results in issues with differences in actual testing locations and stimulus sizes. Using a global score quadrant or hemifield-wise would potentially minimize the effect of this potential variability in testing.

The MATRIX terminates after four presentations while estimating threshold whereas SITA standard crossover checks and retests locations depending on the threshold of neighboring points.[5] This could possibly be the reason for the significantly lesser test duration on the MATRIX than the SITA Standard. Shorter test duration has the potential of improving the reliability parameters arising due to patient fatigue, thereby providing reliable results.

Previous studies have shown that the PSD on the FDT Full threshold N30 test and the SITA Standard have poor correlation, while the MD had good similarity with white on white perimetry.[2,15,17,18] In contrast to this, in our study the Humphrey MATRIX shows good correlation with SITA Standard with regard to MD and PSD. One possible explanation for the difference in results is that the distribution and loss of magno and parvo cellular ganglion cells need not parallel each other as both may be damaged differentially in disease.[15] Another possibility is that the larger stimulus size on the older FDT full threshold test could result in small localized defects being missed on testing and hence affect the PSD measurement. This could explain why the PSD correlated well between both methods in our study as the Humphrey MATRIX uses a smaller stimulus size. The selection of patients with perimetrically established glaucoma in our study could have also accounted for better correlation of PSD between the two instruments. Medeiros *et al.*, who used a receiver operated curve regression equation on PSD adjusted for other covariates like age, loss of neural retinal rim, reported that MATRIX could detect earlier visual field defects than SITA Standard in patients with early glaucoma.[12] The power of the study to detect a significant correlation between the cumulative defect depths for the superior quadrant was 90%, for the inferior quadrant was 70%. The power for test duration was 100%, for MD and PSD correlation was 99% each. The power to detect a difference in GHT was however only 20%. The sample size used in the study is relatively small and may account for the lack of significant difference noted for GHT. The other limitation to this study is that both eyes of patients with reliable visual fields were included. Since both eyes of a single individual are not really independent this could potentially influence our results.

The MD showed a significant positive correlation between instruments. This is expected as the normative population used to create the normal database shows high retinal sensitivity to any stimulus used for testing and cases with pathology would present with abnormal threshold points and corresponding positive correlation. The MATRIX showed denser defects than the SITA Standard, more in the inferior quadrant. This study was carried out on patients diagnosed to have early and moderate glaucoma, the FDT is known to detect disease earlier than conventional white on white perimetry - this may result in denser visual field defects being seen, especially in early and moderate disease.[2,11,17–19] This could possibly explain the poor correlation in the cumulative defect depth in the inferior quadrant between the two instruments. If the MATRIX was producing denser and larger defects in the inferior quadrant this would influence the machine's interpretation of the GHT. Since the GHT compares the superior and inferior hemifields, an asymmetrical hemifield defect depth would result in abnormal GHT classification. Brusini *et al.*, have compared the percentage of depressed defect points on the whole field of preperimetric and perimetrically defined glaucoma patients on the HFA SITA Standard, Full Threshold N30 and MATRIX. Their results suggest that the MATRIX took a slightly longer time than FDT N30 and it provided more detailed characterization of glaucomatous visual field pattern than FDT N30.[18,19]

The Humphrey MATRIX is a quicker test than SITA Standard. Visual field indices on both tests are similar though there is poor agreement on the GHT.
